# The Role of Deep Hypothermia in Cardiac Surgery

**DOI:** 10.3390/ijerph18137061

**Published:** 2021-07-01

**Authors:** Radosław Gocoł, Damian Hudziak, Jarosław Bis, Konrad Mendrala, Łukasz Morkisz, Paweł Podsiadło, Sylweriusz Kosiński, Jacek Piątek, Tomasz Darocha

**Affiliations:** 1 Upper-Silesian Heart Center, Department of Cardiac Surgery, 40-635 Katowice, Poland; gocot@poczta.onet.pl (R.G.); damhud@gmail.com (D.H.); jbis@gcm.pl (J.B.); lmorkisz@gcm.pl (Ł.M.); 2 Department of Cardiac Surgery, School of Medicine in Katowice, Medical University of Silesia, 40-055 Katowice, Poland; 3 Department of Anaesthesiology and Intensive Care, Medical University of Silesia, 40-752 Katowice, Poland; tomekdarocha@wp.pl; 4 Institute of Medical Sciences, Jan Kochanowski University, 25-317 Kielce, Poland; pablo.epka@gmail.com; 5 Faculty of Health Sciences, Jagiellonian University Medical College, 31-126 Krakow, Poland; kosa@mp.pl; 6 Department of Cardiovascular Surgery and Transplantology, John Paul II Hospital, 31-202 Kraków, Poland; jpiatek@onet.pl

**Keywords:** cardiac surgery, deep hypothermia, cardiac arrest, DHCA

## Abstract

Hypothermia is defined as a decrease in body core temperature to below 35 °C. In cardiac surgery, four stages of hypothermia are distinguished: mild, moderate, deep, and profound. The organ protection offered by deep hypothermia (DH) enables safe circulatory arrest as a prerequisite to carrying out cardiac surgical intervention. In adult cardiac surgery, DH is mainly used in aortic arch surgery, surgical treatment of pulmonary embolism, and acute type-A aortic dissection interventions. In surgery treating congenital defects, DH is used to assist aortic arch reconstructions, hypoplastic left heart syndrome interventions, and for multi-stage treatment of infants with a single heart ventricle during the neonatal period. However, it should be noted that a safe duration of circulatory arrest in DH for the central nervous system is 30 to 40 min at most and should not be exceeded to prevent severe neurological adverse events. Personalized therapy for the patient and adequate blood temperature monitoring, glycemia, hematocrit, pH, and cerebral oxygenation is a prerequisite and indispensable part of DH.

## 1. Introduction

Hypothermia, defined as a decrease in body core temperature to below 35 °C, is a supportive technique in cardiac surgery. Four stages of hypothermia can be distinguished: mild, moderate, deep, and profound. While standard deep hypothermia is defined as a body core temperature below 28 °C, this value is lower in cardiac surgery, below 20 °C (Table 1) [1]. Deep hypothermia (DH) is used not only to improve the technical aspect of surgery but primarily to prevent ischemic injury of the central nervous system and crucial organs. The protective effect of hypothermia is provided mainly by slowing the cellular metabolism and thus decreasing its oxygen consumption and energy demand [2]. Metabolic protection offered by hypothermia enables safe circulatory arrest during cardiac surgical intervention. Despite DH being an “old” technique of organ protection, it remains indispensable in specific types of cardiac surgical procedures.

## 2. History

The first reports of using hypothermia as a therapeutic intervention originate from the Hippocratic school of medicine during the 4th century BC, where it was used to support the treatment of tetanus [3] (Table 2). More recent applications of hypothermia date from the 1950s, when Bigelow, after conducting a series of experiments with surface cooling of dogs, suggested its potential usefulness in clinical practice for the first time [4]. He successfully demonstrated that circulatory arrest for 15 min at 20 °C was safe in dogs. The first successful use of hypothermia during surgery on a human patient was attempted in 1952 by Lewis at the University of Minnesota. The surgical intervention consisted of an atrial septal defect repair in a 5-year-old girl, in whom the systemic venous return was closed for 5.5 min, while the patient was surface cooled to 28 °C degrees Celsius [5,6]. In the same year, Swan performed hypothermia-supported atrial septal defect repair in four patients [7] and reported 100 hypothermia-assisted open heart surgeries until 1955 [8]. In 1955, Cooley used DH for brain protection during a first attempt to replace the aortic arch [9]. The first concomitant use of surface cooling and extracorporeal circulation was reported in 1958 by Sealy, Brown, and Young [10]. Deep hypothermia with circulatory arrest was introduced into clinical practice by Dubost in 1960 [11]. In later reports, Mohri from the University of Washington described the technique of surface cooling to 20 °C [12]. In 1963, Barnard and Schrire were the first to apply DH and extracorporeal circulation successfully to surgery of the ascending aorta and aortic arch aneurysm [13]. Later, in 1972, Barratt-Boyes from New Zealand published a modification of the surface cooling technique, proposed initially by Mohri, which enabled open heart interventions with circulatory arrest and subsequent rewarming during extracorporeal circulation [14]. Although the first reports on the use of deep hypothermic cardiac arrest (DHCA) with cardiopulmonary bypass (CPB) appeared in the 1960s, it was not until 1975 that Griepp proved the safety of this technique in aortic arch surgery [15].

As of today, CPB is the crucial element for most cardiac surgical procedures. Regardless of cannulation sites or circuit arrangement, CPB provides organ perfusion during cardiac arrest and control of the circulating blood temperature, thereby allowing regulation of the patient’s body core temperature. Since the first single reports on the use of DHCA, numerous randomized studies have allowed for standardization of the DHCA technique for cardiac surgical procedures. This has translated into good clinical outcomes and increased the reproducibility of DH-assisted surgeries.

## 3. Indications for the Use of DH in Cardiac Surgery

**Figure 1:**
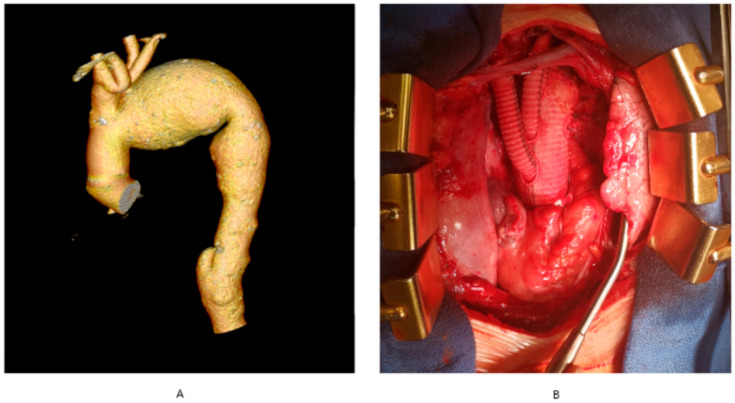
Aortic arch aneurysm. (**A**) computerized tomography angiography of the thoracic aorta with aortic arch aneurysm, (**B**) total arch replacement involving aortic arch replacement with concomitant re-implantation of arch vessels. The authors’ own archives.

Developments in surgical techniques and advances in myocardial protection and extracorporeal circulation have resulted in a substantial narrowing of indications for DH in cardiac surgery. In adult cardiac surgery, DH is currently used both in elective and emergent interventions. Elective interventions include:

- complex aortic arch surgery (Figure 1A,B)
- chronic type-A aortic dissection
- pulmonary embolism surgery
- complex thoraco-abdominal aneurysm surgery
- surgery with co-existing massive calcifications of the ascending aorta precluding cross-clamping (porcelain aorta).

Emergent interventions include:

- surgery for acute type-A aortic dissection

Infant cardiac surgery is the exception to the rule, as hypothermia represents the mainstay of cerebral protection in most interventions for congenital heart defects.

Accordingly, in infant cardiac surgery:

- mild hypothermia is used for corrections of simple defects, where circulatory arrest is not required, and cardiac arrest does not exceed 20 min,
- moderate hypothermia without circulatory arrest is used in older infants with a bodyweight above 10 kg and a complex heart defect, in whom the cardiac arrest duration needed for correction exceeds 20 min,
- DH is used to correct congenital heart defects in infants below 10 kg in bodyweight, and selected older infants.

In infant cardiac surgery, DH still represents the gold standard in procedures involving aortic arch reconstruction, left hypoplastic heart syndrome, and interrupted aortic arch, as well as the multi-stage treatment of infants with a single ventricle in the neonatal period.

## 4. Physiological Effects of Hypothermia

Understanding the role of hypothermia is not a simple matter, as complex systemic and cellular mechanisms mediate its effects. The assumption of benefits from using hypothermia in clinical practice was provided by hypothesizing that the “safe” circulatory arrest period is inversely proportional to one’s body temperature. The term “safe” is reserved for such a period of circulatory arrest, after which no adverse effects can be noted on the structure and function of the internal organs and particularly on the brain, which is most susceptible to ischemia (Table 3).

The brain consumes about 20% of one’s total body oxygen [16]. With a gradual decrease in body temperature, chemical reactions, oxygen consumption, and energy requirements are reduced. The primary protective effect of DH during circulatory arrest at the cellular level consists of the reduction in hydrogen ion concentration. The physical decrease in temperature, water dissociation, and the resulting lower concentration of hydrogen ions significantly slows the biochemical processes, leading to cell death [17]. This is in line with research findings demonstrating that intracellular acidosis occurs first in the brain, subsequently in the heart, and finally in other organs [18]. The human brain is subject to self-regulatory mechanisms, which couple the cerebral blood flow to cerebral oxygen consumption and metabolic activity. This permits safe circulatory arrest in normothermia for only 5 min, after which irreversible changes in brain tissue occur. In his study, Mezrow found that lowering body temperature by 10 degrees Celsius decreases cerebral metabolic activity four-fold and thus increases the tolerance of ischemia [19]. These findings were confirmed in further animal studies [20], where the decrease in cerebral metabolic activity was shown at specific temperatures:

- 28 °C—50%
- 18 °C—19%
- 8 °C—11%

Based on McCullough’s studies [2], safe periods of circulatory arrest in humans for specific temperatures were established:

- 15 °C—30 min
- 10 °C—40 min

Other study findings indicate that although the period of 40 min in deep hypothermic circulatory arrest is “safe”, exceeding this limit is associated with a substantially elevated risk of neurological adverse events [21,22]. In addition to the effect on reducing metabolic requirements, other mechanisms of hypothermia may be relevant, such as suppression of free radicals, inhibition of destructive enzymatic reactions, and inhibition of the biosynthesis, release, and uptake of excitatory neurotransmitters [23,24,25]. Through these mechanisms, hypothermia provides a favorable balance between oxygen supply and demand, slows the onset of ischemic depolarization, decreases the release of ischemia-induced intracellular calcium influx, and suppresses nitric oxide synthase activity.

## 5. The Rules of Cooling and Rewarming

There are several techniques for cooling and re-warming the patient’s body when DH-assisted cardiac surgery is performed. Different cardiac centers have their own protocols in that regard. Nevertheless, some key rules should be followed.

Firstly, the difference between the temperatures of the blood flowing from and to the patient should not exceed 10 °C [26]. Secondly, the cooling process should last at least 30 min, with some authors advocating even 75 min of cooling [27]. Excessive shortening of this time results in rapid temperature swings within the internal organs, which can cause microtrauma injury. Thirdly, during deep hypothermic cardiac arrest, topical head cooling with ice packs is proposed to eliminate passive rewarming [28].

After completing the part of the surgery that requires DH, the rewarming process is started. This is probably the most important stage of DH implementation. The initial rewarming period is associated with flushing out the metabolites and free radicals produced during the circulatory arrest. Similar to cooling, several basic rules should be followed when rewarming. Firstly, the rewarming time should be at least twice as long as the cooling duration [29]. Therefore, this process can take more than 90 min. Secondly, the temperature of the blood flowing to the patient should not exceed 37 °C, in order to ascertain the optimal conditions for regeneration of the central nervous system [30]. The temperature of the central nervous system should not exceed 36.5 °C as hyperthermia amplifies all neurological injuries [30]. Thirdly, continuous monitoring of the patient’s cerebral activity is paramount during rewarming to identify the necessity of immediate therapeutic intervention involving deep anesthesia and sedation.

## 6. Neuroprotection during DH

**Figure 2:**
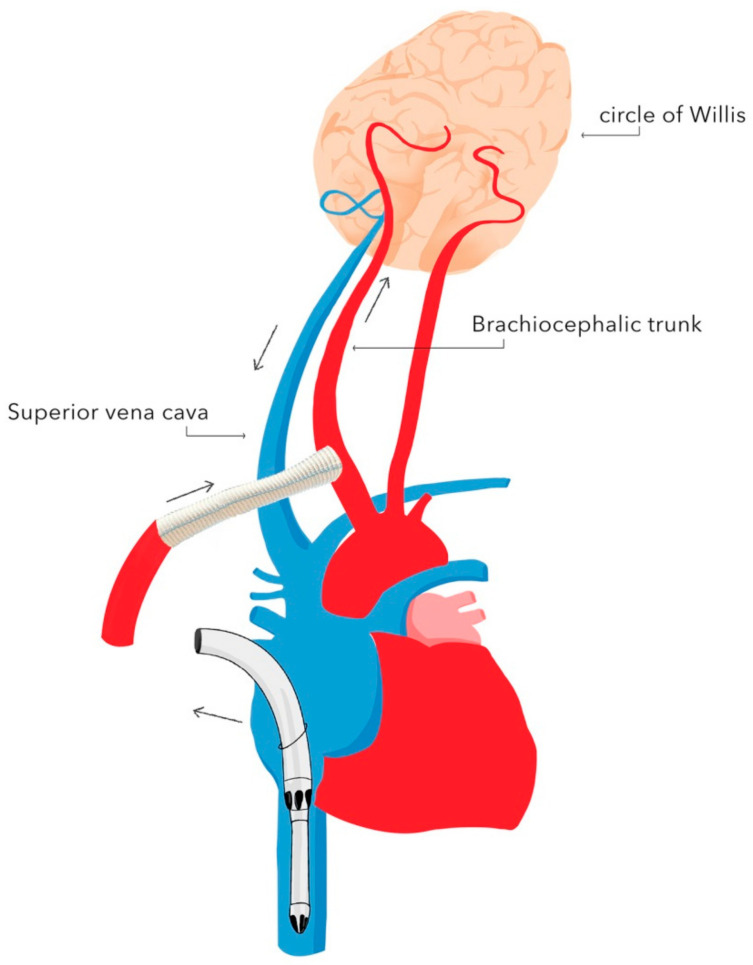
Selective unilateral antegrade cerebral perfusion (unilateral SACP) with brachiocephalic trunk cannulation. Authors’ own graphic.

**Figure 3:**
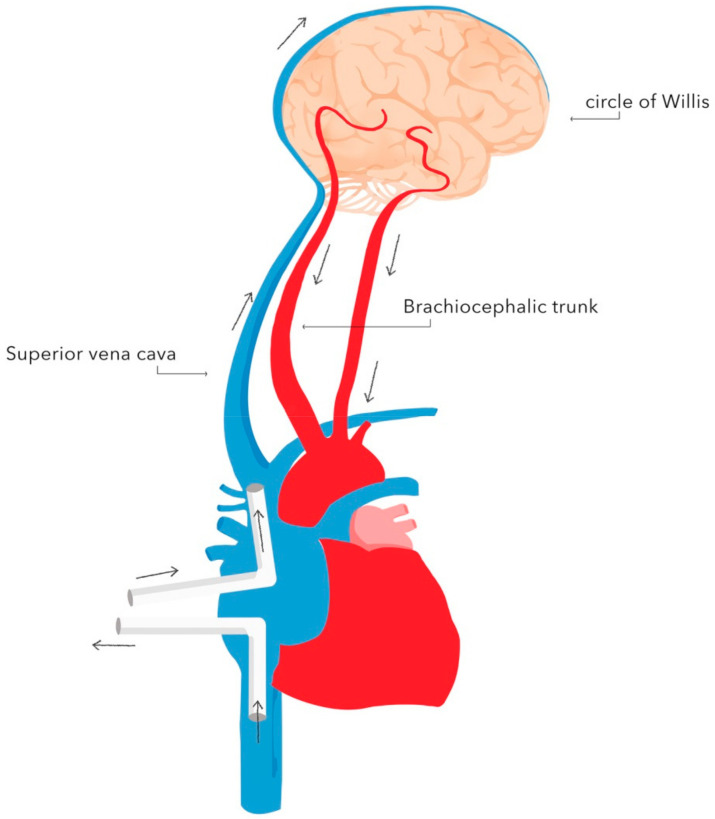
Diagram of retrograde cerebral perfusion (RCP). Oxygenated blood is delivered through the superior vena cava, whereas deoxygenated blood is returned through the cerebral arteries. Authors’ own graphic.

The basic techniques of neuroprotection supporting DH during circulatory arrest include:

- selective antegrade cerebral perfusion (SACP) (Figure 2) or retrograde cerebral perfusion (RCP) (Figure 3);
- cerebrospinal fluid drainage;
- pharmacotherapy.

Currently, SACP represents the most frequently used technique of neuroprotection during DH. There are two types of SACP: unilateral and bilateral [31,32]. Bilateral SACP provides blood supply to the brain through both the left and right carotid arteries by cannulating the brachiocephalic trunk and the left carotid artery. Unilateral SACP provides blood supply to the brain through one of the carotid arteries. In this technique, the continuity of the arterial circle of Willis is crucial, as it ensures blood supply to both brain hemispheres. In most cases, an 8 mm Dacron graft is anastomosed to the brachiocephalic trunk. Alternatively, the right subclavian artery or the left carotid artery can be used as vascular access for the blood supply. During SACP, the flow is reduced to 10–20 mL/kg body weight/min, and the pressure in the right radial artery is maintained at 40–50 mmHg [33]. SACP is preferred as it provides continuous blood supply to the brain throughout surgery, which enables one to accept higher body temperatures in the range of 25–26 °C—the duration of cooling and rewarming are shortened—and consequently, the CPB duration and CPB-associated adverse events are reduced [34]. However, the research failed to demonstrate a reduction in the stroke rate in patients in whom SACP was used [35]. The reported rate of neurological adverse events ranges from 1% to 16% [36,37].

Retrograde cerebral perfusion (RCP) consists of brain perfusion by reversing blood flow in the cerebral vessels. The brain is supplied retrogradely—oxygenated blood is delivered through the superior vena cava, and deoxygenated blood returns through the cerebral arteries. This method of perfusion is possible as there are no valves in the cerebral venous system. A perfusion pressure of 25 mmHg is recommended during RCP. The implementation of RCP enables safe prolongation of circulatory arrest during DH to 60 min [38]. The advantages of RCP include maintaining a constant brain temperature during DH and flushing out air emboli and toxic metabolites [39]. A reduced rate of neurological adverse events associated with RCP compared with standard DH has been demonstrated [40,41,42].

Another neuroprotective intervention during DHCA in complex interventions involving the descending aorta is cerebrospinal fluid drainage. The earliest reports come from animal studies performed during the 1960s [43]. The most beneficial effect of cerebrospinal fluid drainage is reducing pressure in the spinal canal, thus decreasing the forces pressurizing the spinal cord [44]. A drainage catheter is placed prior to surgery (before administering anticoagulants and antiplatelet agents) in the 2nd or 3rd lumbar space and remains in place for 2–3 days after surgery. It is used to monitor the pressure in the spinal canal and, if required, to drain the cerebrospinal fluid passively or actively in order to reduce the pressure. The recommended target values for cerebrospinal fluid pressure are 8–10 mmHg during surgery and 10–12 mmHg in the postoperative period [44].

Neuroprotective pharmacotherapy includes several groups of medications that are used during DH:

- Anesthetics agents.
- Steroids.
- Other drugs.

As studies regarding the benefits of general anesthetics remain inconsistent, the choice of anesthetic agent depends mainly on the anesthesiologist’s personal preferences. It has been suggested that propofol—and opioid-based anesthetics—decrease cerebral metabolism without affecting cerebral flow–metabolism coupling, which may occur during the use of volatile agents [45]. In contrast, data from animal studies suggest that the use of desflurane may improve the neurological outcome after DHCA [46]. However, it should be noted that the solubility of gases and volatile agents in the blood increases at low temperatures, which may significantly increase the time to achieve adequate partial pressure in the central nervous system. In addition, rewarming from hypothermia can significantly increase the partial pressure of inhaled anesthetics, and thus alter the depth of anesthesia. An intravenous anesthetic that significantly affects brain metabolic activity by inducing burst suppression is thiopental. Its beneficial effects include reducing energy requirements for synaptic transmission and preventing focal brain injury [47,48,49]. However, it should be remembered that thiopental has a negative inotropic effect, which can impact the early postoperative course.

Although there is still inconclusive evidence of a neuroprotective effect of corticosteroids, their use during DHCA should be considered. Steroids reduce the systemic inflammatory response syndrome (SIRS), which can be triggered by CPB [50]. Moreover, they reduce the concentration of inflammatory cytokines [51]. Some studies indicate that the use of steroids after DH may have a beneficial effect on cerebral metabolism, enhance brain tissue blood perfusion, and reduce capillary permeability, thus preventing tissue edema [51,52]. Corticosteroids should be initiated at least several hours before surgery in order to be effective.

The use of other potentially neuroprotective drugs, such as calcium channel blockers, aprotinin, nafamostat, mannitol, deferoxamine, magnesium, lidocaine, fosphenytoin, and thromboxane A2 receptor blockers, is still a matter of debate and clinical research.

## 7. Monitoring during DH

During DH, outside of standard monitoring, several additional parameters should be monitored.

### 7.1. Physical

- temperature at the arterial outlet of the oxygenator (a surrogate for cerebral perfusate temperature) and two anatomical sites (nasopharyngeal cavity, tympanic membrane, bladder, esophageal or rectal);
- arterial blood pressure at three different sites (right radial artery, left radial artery, femoral artery);
- assessing the function of the central nervous system:
  ◦ electric: electroencephalogram (EEG) or somatosensory evoked potentials (SSEP);
  ◦ oxygen delivery: jugular venous bulb saturation (JVBS), near-infrared spectroscopy (NIRS), transcranial doppler sonography.

As a result of the thermal inertia of human body compartments, during DHCA, temperature measurements from the bladder or rectum may differ by up to several degrees Celsius from the cerebral temperature. After equilibration of the core temperature, bladder temperature is a recommended method to monitor body core temperature. Temperature measurement from the nasopharyngeal cavity or esophagus is well correlated with brain temperature. In order to minimize the risk of cerebral injury during rewarming from DHCA, it is recommended to maintain an arterial blood outlet temperature below 37 °C [26].

At a temperature below 28 °C, there is a gradual slow down to complete suppression of EEG, allowing assessment of the effectiveness of cooling before DHCA. Although this is a sensitive indicator of neuronal damage, performing and interpreting an EEG is challenging and currently rarely practiced.

Evoked potential monitoring techniques are based on the analysis of responses to a visual or auditory stimulus (somatosensory evoked potentials, SSEPs) or a motor stimulus (motor evoked potentials, MEPs). The suppression of the evoked potential components is used as a surrogate of adequate cooling before DHCA [53,54].

Continuous fiberoptic jugular venous blood saturation (SjO2) monitoring provides an estimation of the overall balance between brain oxygen supply (VO2) and demand (DO2). SjO2 monitoring is used to assess the adequacy of brain cooling before DHCA. A low SjO2 before DHCA suggests increased brain activity [55].

Currently, the most popular and frequently used method for central nervous system functional assessment is near-infrared spectroscopy (NIRS). As near-infrared light penetrates the skull bones, by placing the NIRS source and detector on the patient’s forehead, it is possible to assess the oxygenation of the cerebral cortex (rSO2) of the frontal cortex. Whereas SjO2 provides an assessment of overall central nervous system oxygen consumption, NIRS techniques offer an insight into local brain tissue oxygenation.

The typical rSO2 range is 55–80%, while a drop below 50% absolute or 20% from baseline values indicates the need for intervention [56]. Decreased rSO2 may be suggestive of CPB cannula misplacement [57]. Nevertheless, there is still no clear evidence that interventions that improve rSO2 saturation prevent stroke or postoperative cognitive disorders (COPD) [58].

Transcranial Doppler ultrasonography (TCD) may be used to measure cerebral blood flow (CBF) and detect microemboli, both solid and gaseous. However, the change in blood flow velocity through the MCA may not correlate with the change in CBF, especially in laminar CPB flow [59]. In addition, it should be noted that there is currently no conclusive evidence for the correlation of POCD and the intraoperative detection of microemboli [60].

### 7.2. Biochemical

- Blood glucose concentration—DH induces hyperglycemia, which is amplified by administering steroids during circulatory arrest. There are two mechanisms of the adverse impact of hyperglycemia: increased intracellular acidosis which enhances apoptosis, and triggering the release of amino acids that adversely affect the ischemic nervous tissue [61]. Therefore, a restrictive approach to blood glucose concentrations below 180 mg/dL is recommended during DH [62].
- Hematocrit—although not routinely practiced, hemodilution to a hematocrit of 20% is assumed to improve flow in the microcirculation, while values of 30% may be beneficial [63]. During rewarming, under normothermic conditions, hemodilution can significantly impair the oxygen flow to the tissues and may be associated with hemostasis impairments. [64,65].
- Arterial blood gas analysis—in hypothermia, the solubility of carbon dioxide increases, resulting in a decrease in the partial pressure of CO2 and thus alkalosis. Two different approaches to achieving an acid–base balance during hypothermia have been developed. The pH-stat perfusion strategy involves measuring pH and pCO2 corrected to the patient’s current (low) temperature and maintaining a pH of 7.4 and pCO2 of 40 mmHg during the surgery. The alpha-stat strategy is to keep the pH at 7.4 and pCO2 at 40 mmHg by measuring these values from a blood sample heated to 37 °C. In order to preserve cerebral self-regulation and intracellular electrochemical neutrality in patients in induced mild/moderate hypothermia, the use of an alpha-stat strategy appears to be acceptable [66,67]. However, for induced circulatory arrest in deep hypothermia, the pH-stat strategy should be preferred to maximize brain protection, enhance cerebral blood flow, cerebral oxygenation, and improve brain cooling [68,69,70].

## 8. Advantages and Drawbacks of DH

### 8.1. Advantages

- enables complex surgical interventions involving the aortic arch during circulatory arrest;
- indispensable for pulmonary embolism surgery;
- in infant congenital defect surgery, it improves the technical aspect, as the CPB cannulas limiting the operating field can be temporarily removed during circulatory arrest;
- protects the tissues (particularly nervous tissue) from ischemic damage by reducing the cellular metabolism.

### 8.2. Drawbacks

- as DH does not stop the cellular metabolism completely, the time of its application is limited;
- DH requires advanced monitoring of physical and biochemical parameters;
- Risk of excessive bleeding due to coagulopathy;
- to limit the rate of neurological adverse events, the use of neuroprotective measures such as pharmacotherapy and selective organ perfusion techniques is necessary.

## 9. Future Perspectives

Therapeutic hypothermia has become one of the indispensable tools for cardiac surgeons. The open complex aortic surgery, including the ascending aorta, aortic arch, descending aorta, and thoracoabdominal aorta regardless of the pathology (aneurysm, dissection, intramural hematoma), requires cardiac arrest with organ protection provided by deep hypothermia or isolated cerebral perfusion with moderate hypothermia [71,72]. Currently, there is no conclusive evidence indicating the superiority of one of these two methods, which should encourage further research [73]. Surgical corrections of congenital heart disease performed in children and adults owe their good and reproducible results, among others, to the use of hypothermia [74].

Due to the increasing popularity of transcatheter aortic valve implantation (TAVI), it may seem that surgical aortic valve replacement (SAVR) in patients with massive calcifications of the ascending aorta and inability to cross-clamp the aorta is no longer justified. However, Urbanski et al. showed that complex aortic valve surgery and removing the calcified ascending aorta performed under therapeutic hypothermia does not significantly increase mortality compared to TAVI but notably reduces neurological adverse events [75].

In the COVID-19 pandemic, thromboembolic incidents have been observed more frequently, and treatment may require invasive procedures. Regardless of the cause, pulmonary thrombectomy surgery is performed by choice with cardiac arrest in deep hypothermia [76,77,78].

Additionally, the number of patients requiring cardiac reoperations is constantly increasing. The high risk of complications is related to the potential damage to mediastinal structures (including the primary coronary artery bypass grafts), bleeding, infection, cardiac tamponade, and sternal instability. The use of combined techniques involving deep hypothermia with low-flow CPB and ventricular fibrillation avoids both the aortic and the LIMA bypass cross-clamping. This approach is being used irrespective of the surgical access technique (resternotomy or right thoracotomy with videothoracoscopy) and the type of the procedure [79,80].

In cardiac surgery, hypothermia combined with cold cardioplegia (Custodiol) also has an important protective effect in complex procedures requiring prolonged aortic cross-clamping, or procedures in which frequent administration of cardioplegia is difficult because of the access used [81].

Furthermore, the use of deep hypothermia with a cardiac arrest can help to manage complications that occur during surgery (repair of the damaged aorta, pulmonary artery, right or left ventricle, or superior and inferior vena cava).

Although nearly seventy years have passed since hypothermia was first used during the surgery [6], it is still one of the fundamentals of cardiac surgery, allowing a wide range of procedures to be performed safely.

Despite ongoing efforts to optimize and standardize the therapeutic hypothermia technique, it still requires further research.

## 10. Conclusions

DH is a mainstay technique enabling the performance of complex surgeries. Despite its 70-year history, it is still being developed and enhanced with additional solutions such as selective brain perfusion or retrograde brain perfusion, which improve outcomes. However, it should be remembered that the period of circulatory arrest in DH considered safe for the central nervous system is 30 to 40 min at most and should not be exceeded in order to prevent severe neurological adverse events. Implementing patient personalized surgery with adequate blood temperature monitoring, glycemia, hematocrit, pH, and cerebral oxygenation is a prerequisite and indispensable part of DH.

## Figures and Tables

**Table 1:** Classification of hypothermia.

Stages	Body Core Temperature	
	Standard Values	Cardiac Surgery
Mild (°C)	35–32	34–28.1
Moderate (°C)	31.9–28	28–20.1
Deep (°C)	27.9–20.1	20–14.1
Profound (°C)	≤20	≤14

**Table 2:** History of hypothermia in cardiac surgery.

Name	Year	Development
Hippocrates	4th century BC	Hypothermia used to support the treatment of tetanus
Larrey	1812	Local hypothermia used to alleviate the pain during amputations of extremities
Bigelow	1950	Safe circulatory arrest in dogs for 15 min at a temperature of 20 °C
Lewis	1952	First successful surgery in human patient with the use of hypothermia
Swan	1955	100 open heart surgeries with the use of hypothermia
Cooley	1955	Use of deep hypothermia for cerebral protection during aortic arch surgery
Sealy, Brown, Young	1958	Clinical use of concomitant surface cooling and cardiopulmonary bypass
Dubost	1960	Deep hypothermia with circulatory arrest
Mohri	1963	Technique of surface cooling to 17–20 °C
Barnard, Schrir	1963	Successful use of deep hypothermia and cardiopulmonary bypass during ascending aorta and aortic arch surgery
Barratt-Boyes	1972	Technique of surface cooling enabling open heart correction with circulatory arrest and subsequent re-warming in extracorporeal circulation

**Table 3:** Pathophysiology of hypothermia.

Symptoms	Mild	Moderate	Deep
Neuro-muscular	ataxia dysarthria shivering	stiffness of muscles and joints	muscle contraction
Neurological	confusion amnesia apathy limited awareness	limited consciousness	dilated pupils coma loss of self-regulation
Circulatory	tachycardia vascular constriction blood pressure increase	bradycardia widening of QRS complexes elevation/depression of ST segment T-wave inversion AV block QT segment prolongation	serious bradycardia asystole ventricular fibrillation
Respiratory	tachypnae HbO_2_ curve shifts to the left	bradypnae bronchial constriction	lactic acidosis HbO_2_ curve shifts to the right

## Data Availability

Not applicable.
